# Dual effects of entomopathogenic fungi on control of the pest *Lobesia botrana* and the pathogenic fungus *Eutypella microtheca* on grapevine

**DOI:** 10.1186/s40659-021-00367-x

**Published:** 2021-12-24

**Authors:** Juan Aguilera-Sammaritano, Juan Caballero, María Deymié, Melisa Rosa, Fabio Vazquez, Delia Pappano, Bernardo Lechner, Marcia González-Teuber

**Affiliations:** 1 Departamento de Química Ambiental, Facultad de Ciencias, Universidad Católica de la Santísima Concepción, Concepción, Chile; 2 Instituto de Biotecnología, Facultad de Ingeniería, Universidad Nacional de San Juan, San Juan, Argentina; 3 Instituto de Ciencias Básicas, Facultad de Ingeniería, Universidad Nacional de San Juan, San Juan, Argentina; 4 Instituto de Micología y Botánica, Facultad de Ciencias Exactas y Naturales, Universidad de Buenos Aires, Buenos Aires, Argentina

**Keywords:** Entomopathogenic fungi, European grapevine moth, *Eutypella microtheca*, Grapevine, Ligninolytic fungus, *Lobesia botrana*

## Abstract

**Background:**

Entomopathogenic fungi (EPF) are the natural enemies of insect pests. Nevertheless, research on the use of EPF for simultaneous prevention of pest and disease agents on the same crop is limited. In this study, we explored the potential dual effects of three strains of the EPF *Metarhizium anisopliae* on the control of detrimental agents of *Vitis vinifera* L., including different developmental stages (larvae, pupae, and adult) of the insect pest *Lobesia botrana* and the phytopathogenic fungus *Eutypella microtheca*.

**Methods:**

Laboratory pathogenicity trials were performed to examine the effects of the three *M. anisopliae* strains on the mortality rate of *L. botrana*. In addition, field trials were conducted to assess the biocontrol potential of one selected *M. anisopliae* strain on the larval stage of *L. botrana*. Moreover, inhibitory effects of the three EPF strains on *E. microtheca* growth were examined in vitro.

**Results:**

All the *M. anisopliae* strains were highly effective, killing all stages of *L. botrana* as well as inhibiting the growth of *E. microtheca*. The in vitro mortality of larvae treated with the strains was over 75%, whereas that of treated pupae and adults was over 85%. The three EPF strains showed similar efficacy against larvae and adult stages; nevertheless, pupal mortality was observed to be strain dependent. Mortality of *L. botrana* larvae ranged from 64 to 91% at field conditions. Inhibition of *E. microtheca* growth reached 50% in comparison to the control.

**Conclusions:**

Our study showed that *M. anisopliae* strains were highly effective in ensuring control of two different detrimental agents of *V. vinifera* L., providing new evidence to support the dual effects of entomopathogenic fungi.

## Background

The grapevine (*Vitis vinifera* L.) is one of the most cultivated fruit crops worldwide, with a cultivated area of 7.4 million ha. However, grapevine production is affected by several pests and diseases, resulting in high use of pesticides and chemicals [1–4]. Among the numerous pests and diseases of grapevine, the European grapevine moth (*Lobesia botrana* Den. and Schiff.) and the ligninolytic fungus (*Eutypella microtheca* Trouillas, W.M. Pitt and Gubler) are of considerable economic importance to vine production worldwide [5].

*Lobesia botrana* is one of the most relevant pests of vineyards [6]. Although this pest is endemic to the Palearctic Region [7], is economically more important in southern Europe (France, Spain, Italy and the Mediterranean islands) and South America (Argentina and Chile) [8–11], where can cause substantial crop losses from larvae feeding on young flower buds and fruit [12, 13]. Additionally, ligninolytic fungi, including *E. microtheca*, *Phaeomoniella chlamydospora*, and the genus *Phaeoacremonium* [5, 14, 15], are responsible for pathogenic symptoms, such as wood necrosis and/or discolouration, vascular infections, and decay, which are commonly known as grapevine trunk diseases [16]. These two biological agents are highly detrimental to vine production worldwide. While *L. botrana* is one of the most injurious insect pests of table and wine grape production [17], the economic costs caused by the effects of ligninolytic fungi on grape production is estimated to be more than 1.5 billion dollars per year [18].

Conventional methods of preventing and controlling *L. botrana* and ligninolytic fungi involve the use of chemical compounds [15, 19, 20]. Additionally, the management and prevention of *L. botrana* in vineyards involve mating disruption [21–24]. However, these methods have been ineffective, as this pest continues to proliferate in vineyards. Moreover, the widespread use of chemical products in controlling crop pests and diseases have been widely criticised because of their negative impacts on biodiversity and human health [25, 26]. To the best of our knowledge, researchers are yet to develop an effective, economic, and eco-friendly method for preventing and managing *L. botrana* infestation and *E*. *microtheca* infection. Owing to the negative impacts of synthetic pesticides on the environment, biopesticides such as entomopathogenic fungi (EPF), have been identified as a promising alternative for environmentally friendly pest management programmes [27].

EPF are a group of environmentally safe fungi used for biological control of insects and other arthropod pests of agricultural crops. They comprise a wide range of morphologically, phylogenetically, and ecologically diverse fungal species [28]. A wide range of EPF species have been established as important biocontrol agents in many natural and artificial ecosystems [29], to defend against a large number of insect species [30]. Additionally, recent studies suggest that EPF possess inhibitory actions against some phytopathogenic fungal species [31–34]. Thus, there is growing interest in the use of these organisms in integrated pest management (IPM) programmes [35]. However, research on the use of EPF in the simultaneous prevention and management of several pest and disease agents of the same crop is limited.

Here, we examined the effectiveness of three different strains of the EPF *Metarhizium anisopliae* in controlling *L. botrana* at both laboratory and field conditions, as well as growth of *E. microtheca *in vitro. Therefore, the findings of this study serve as a basis for the use of EPF in pest and disease control providing new evidence for the inclusion of EPF as dual biocontrol agents in vineyards.

## Methodology

### Biological material

#### Entomopathogenic fungal strains

EPF were obtained from soils under vine crops in San Juan, Argentina (31° 65′ 67″ S; 68° 58′ 51″ W). Specimens were isolated from soil samples using the *Tenebrio molitor* larval baiting technique [36]. Three *M. anisopliae* strains (Metsc.) Sorok. (CEP413, CEP589 and CEP591) were selected for trials based on preliminary inhibitory tests against *L. botrana* [32]. The strains were first identified morphologically [37] and then genetically [38]. The *M. anisopliae* strains showed cylindrical conidia (between 5 and 9 µm of length), with an olive-green colouring, which is characteristic of the species. The isolated EPF were preserved in the Fungal Entomopathogens Collection of the ‘Centro de Estudios Parasitológicos y de Vectores’ (CEPAVE-CONICET, La Plata, Buenos Aires, Argentina).

#### Lobesia botrana specimens

The effects of *M. anisopliae* strains against the fifth instar larvae (L_5_), pupae (P_p_), and adults (A_d_) stages of *L. botrana* were examined. Newly emerged larvae were obtained from a breeding colony in Mendoza, Argentina (33° 01′ 52″ S, 68° 46′ 34″ W). Fifth instar larvae of *L. botrana* were selected for trials based on previous studies reporting that damage by *L. botrana* increases with larval age [39]. The larvae were reared on an artificial diet [40], which was provided ad libitum during the complete growth period. Individuals from all stages were maintained in a growth chamber under a 16-h photoperiod, at a temperature of 25 ± 5 °C, and relative humidity ranging from 30 to 50%. This procedure has been reported to be effective in producing large numbers of larvae [41].

#### Phytopathogenic fungal strain

The phytopathogenic fungal strain was obtained from a vine culture (31° 76′ 10″ S, 68° 57′ 94″ W) located in San Juan Province, Argentina. Fourteen plants displaying clear symptoms of eutypiosis, including chlorotic and small leaves as well as short, rough internodes, stunted shoots, and dark brown wood with hard wedge-shaped consistency [42], were selected from the field. The infected plants were uprooted, and infected tissues were excised and taken to the laboratory. We collected 2–5 subsamples from each symptomatic wood fragment. To remove external non-parasitic microorganisms, the surface of each subsample was sterilised as follows: 30 s in 70% ethanol, 2 min in 3.5% NaOCl, and 30 s in 70% ethanol [43]. The subsamples were then cultured in Petri dishes containing malt extract agar medium (MEA) and on plates containing potato glucose agar (PGA) (Britania®). Both MEA and PGA culture media were supplemented with 100 mg/mL of streptomycin sulphate, 50 mg/mL of chlortetracycline HCl, and 5 mg/mL of dichloran to prevent bacterial and yeast contamination [44]. Plates were immediately cultured in the dark in a growth chamber at 25 °C and inspected daily over a period of 4 weeks until fresh mycelia were observed. Subsequently, successive isolations were performed to derive pure cultures of the fungal strain.

The identification of the phytopathogenic fungus was based first on morphological observations, followed by molecular identification [45]. Genomic DNA was extracted from the mycelium growing on liquid Peptone Malt (MP) medium at 21 days using a DNeasy UltraCkean Microbial Kit (Qiagen®, Germany), following the manufacturer’s protocol. Then, the extracted DNA was purified, and the internal transcribed spacer (ITS) region of the complete rDNA (ITS1, ITS2 and 5.8S) was amplified by PCR. The PCR products were sent to Macrogen Inc^©^ (Seoul, Korea) for purification and sequencing. The strain CC58 was identified as *E. microtheca* (SINAVIMO #9287) and preserved in the Centro de Investigación y Extensión Forestal Andino Patagónico (CIEPFAP) mycological collection, Chubut, Argentina.

### EPF control on *L. botrana* in vitro

The evaluation of EPF efficacy in vitro and in vivo can be achieved using different application techniques, such as dipping insects in a spore suspension [46, 47], using topical micro-applications [48–51], or spraying EPF formulations directly to the insects or plants [52, 53]. In this study, EPF pathogenicity across different stages of *L. botrana* was tested using a micro-application technique, which ensures a higher level of precision, accuracy, and reliability of the pest susceptibility [54], and is useful to compare insect susceptibility to EPF among different developmental stages. A laboratory experiment was performed to examine the effects of three EPF strains on the mortality rate of *L. botrana* using 240 fifth instar larvae (L_5_), 240 pupae (P_P_), and 240 adults (A_d_) of *L. botrana* using a topical micro-application technique [50]. EPF were applied to un-cocooned pupae [6, 55, 56]. The experiment comprised four groups: (1) insects treated with EPF strain CEP413, (2) insects treated with EPF strain CEP589, (3) insects treated with EPF strain CEP591, and (4) control group (insects treated with an EPF-free solution). Treatment groups were topically treated [50, 54] with a 1 µL drop (1 × 10^6^ c/mL) of the respective strain using a Hamilton® micro dispenser (7000 Series Syringes®). The control group was treated with 1 µL of distilled water. Mortality rates were measured using a subset of 20 individuals per replicate. After fungal application, the L_5_, P_P_, and A_d_ of *L. botrana* were carefully transferred to sterile 90-mm Petri dishes lined with sterile filter paper (Whatman®). Immediately, dishes were incubated in growth chambers at 27 °C. Considering the high competition for food among larvae [57], an artificial diet [40] was offered ad libitum during the study period. Adults were carefully inoculated immediately after pupal eclosion and transferred to plastic trays (20 cm × 10 cm × 15 cm). The adults were nourished during the experimental period with a 5% ascorbic acid solution provided using a plastic syringe [41]. Since mortality over long time periods is not representative of field conditions, the mortality rates of L_5_, P_p_, and A_d_ of *L. botrana* were measured up to the 7th day of the experiment. Dead European grapevine moths were carefully removed from the Petri dish to avoid horizontal transmission. To prevent adults from escaping from the trays, dead individuals were not removed until the end of the experiment. Abbott’s equation [58] was used to obtain the corrected mortality (CM).

### EPF control on *L. botrana* in the field

Efficacy of EPF on the control of the larval stage of *L. botrana* was tested in the field. Based on the corrected mortality (CM) observed in the laboratory experiments, we selected one *M. anisopliae* strain (CEP591) for field trials. The trial was repeated across different seasons over 1 year (September 2018–March 2019): (S_1_) during spring, when plants displayed well developed inflorescences, (S_2_) during early summer, when plants had undeveloped bunches, and (S_3_) during late summer, which comprised the vine veraison stage. Trials were carried out in an experimental field (“*Las Mellizas*”) located in San Juan, Argentina (31° 58′ 58″ S; 68° 26′ 28″ W), which is planted with *V. vinifera* L. cv. Merlot since 2011. The field trial was performed within an approximate area of 4.63 ha. No chemical or biological insecticides were added to vines 45 days before trials or during trials. To avoid pupation during trials, we performed the field experiments with newly emerged L_3_–L_4_ larvae of *L. botrana*. Trials consisted of two treatments (EPF+ and EPF−), with six replicates each treatment. Micro-application technique was used. For the EPF+ treatment, 20 larvae were topically treated with 1 µL drop (1 × 10^6^ c/mL) of CEP591. The control group (EPF−) were treated with 1 µL of sterile distilled water. After larvae were topically treated, they were immediately brought to the experimental field and carefully placed on selected grapevine bunches in groups of 20 treated individuals per bunch. The grapevine bunches were then immediately covered with a fabric mesh of 500 × 500 μm of pore diameter. Fabric bands were then attached from the top of the mesh to the rachis of each bunch to enclose the experimental unit. This helped to maintain a closed system, preventing the entry or exit of insects into the experimental unit. Treatments were spatially separated by at least 20 m. After 7 days, bunches were carefully removed from plants and transferred to the laboratory in closed plastic containers to determine larvae mortality. Environmental temperature (T°) and relative humidity (RH) during the three seasons were obtained from an automatic weather station (Davis Instruments® – Mod. Vantage pro-2).

### *Eutypella microtheca* growth assessment

We examined the inhibitory rate of the three strains of EPF (CEP413, CEP589, and CEP591) on the growth of *E. microtheca* strain (CC58). CC58 was used in this study because it was the most abundant phytopathogenic strain affecting grapevine plants at the study site (see above for detailed geolocation information). CEP413, CEP589, and CEP591 were co-cultured with CC58 in separate Petri dishes containing potato glucose agar (PGA) media (Britania®). To achieve this, a 5-mm mycelial disc, obtained from the edges of a 10-days-old culture of CC58, was placed at the centre of a dish containing 20 mL of PGA. Immediately, four discs of one EPF strain (from 10-days-old cultures) of the same diameter as those of the CC58 strain were placed carefully at four sites at approximately 3 cm from the centre of the Petri dish [59]. The Petri dishes were inverted to prevent conidia from falling onto the agar medium and then incubated in the dark at 28 ± 2 °C. The growth of the *E. microtheca* colony was measured every 96 h on two perpendicular axes over the following 20 days under a stereomicroscope (Lancet Instruments®, Model ZTX-30Y-C2, China) using a digital calliper. The control was determined by measuring the radial growth of *E. microtheca* strain growing in isolation (agar discs without EPF) on separate Petri dishes (indicative of the potential growth of the *E. microtheca* strain). The percentage of growth inhibition (GI) was calculated with the following formula: GI (%) = ((A − B)/A) × 100, where A represents the radial growth (mm) of the control treatment, and B represents the radial growth (mm) of the pathogen with the EPF setup [60]. Three replicate plates were prepared for each phytopathogenic/EPF strain combination and for the control treatment.

### Statistical analyses

Effects of CEP413, CEP589, and CEP591 on mortality rate of larvae (L_5_), pupae (P_p_), and adults (A_d_) of *L. botrana *in vitro were analysed using a one-way ANOVA (independent factor: EPF strains, response variable: corrected mortality (CM)). Efficacy exhibited by CEP591 over *L. botrana* in the field was analysed using a one-way ANOVA (independent factor: seasons, response variable: CM). Antagonistic effects of EPF on *E. microtheca* growth were also analysed using a one-way ANOVA (independent factor: EPF strains, response variable: pathogen inhibition growth (%). A post hoc Fisher’s LSD test was performed to analyse differences among treatments. All statistical analyses were performed using Infostat® statistical software [61].

## Results

### Biological control of EPF on *L. botrana* in vitro

**Fig. 1:**
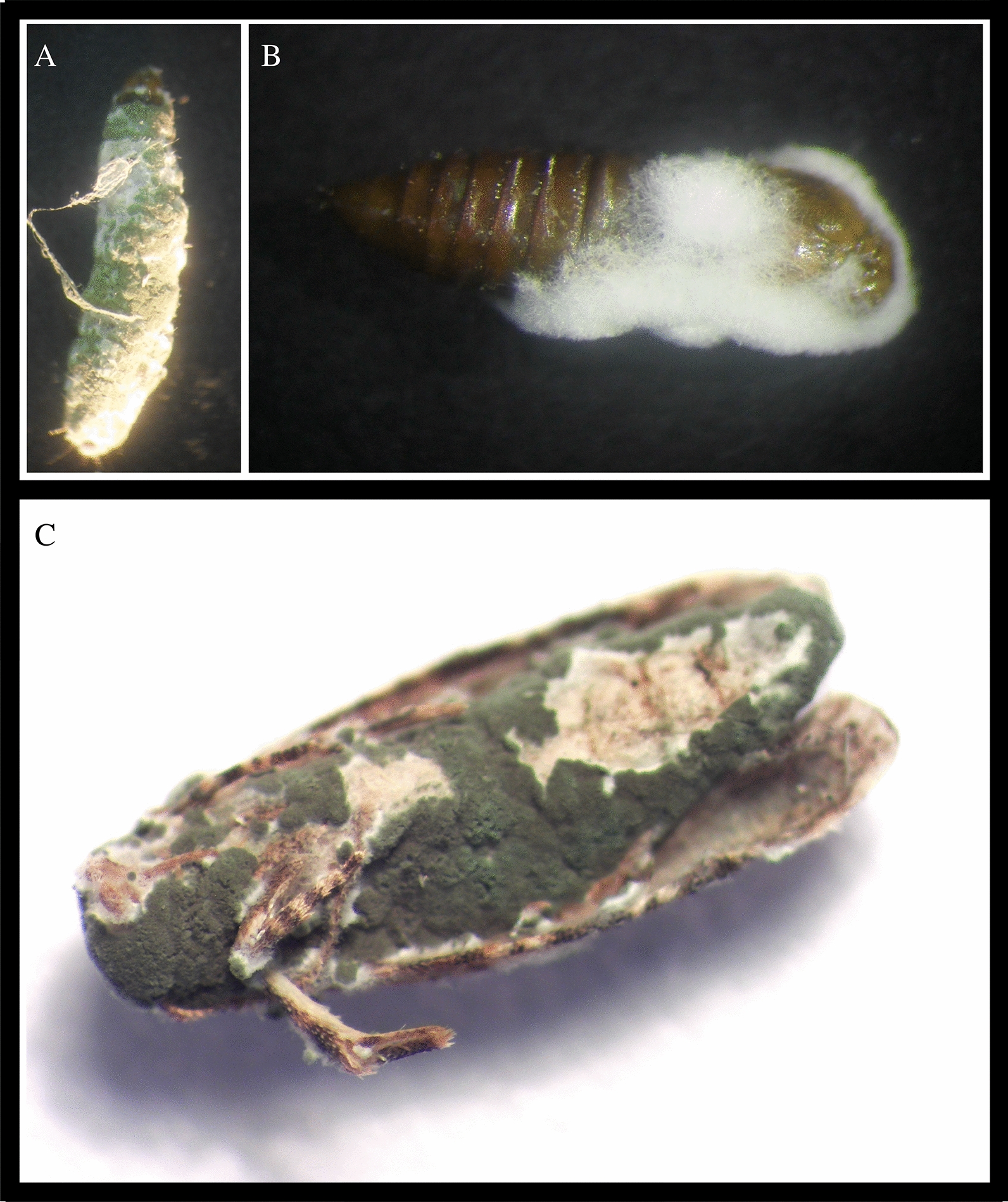
Effects of the EPF *Metarhizium anisopliae* on *Lobesia botrana*. Effective infection of *Metarhizium anisopliae* strains can be observed for larvae (**a**), pupae (**b**), and adults (**c**)

**Fig. 2:**
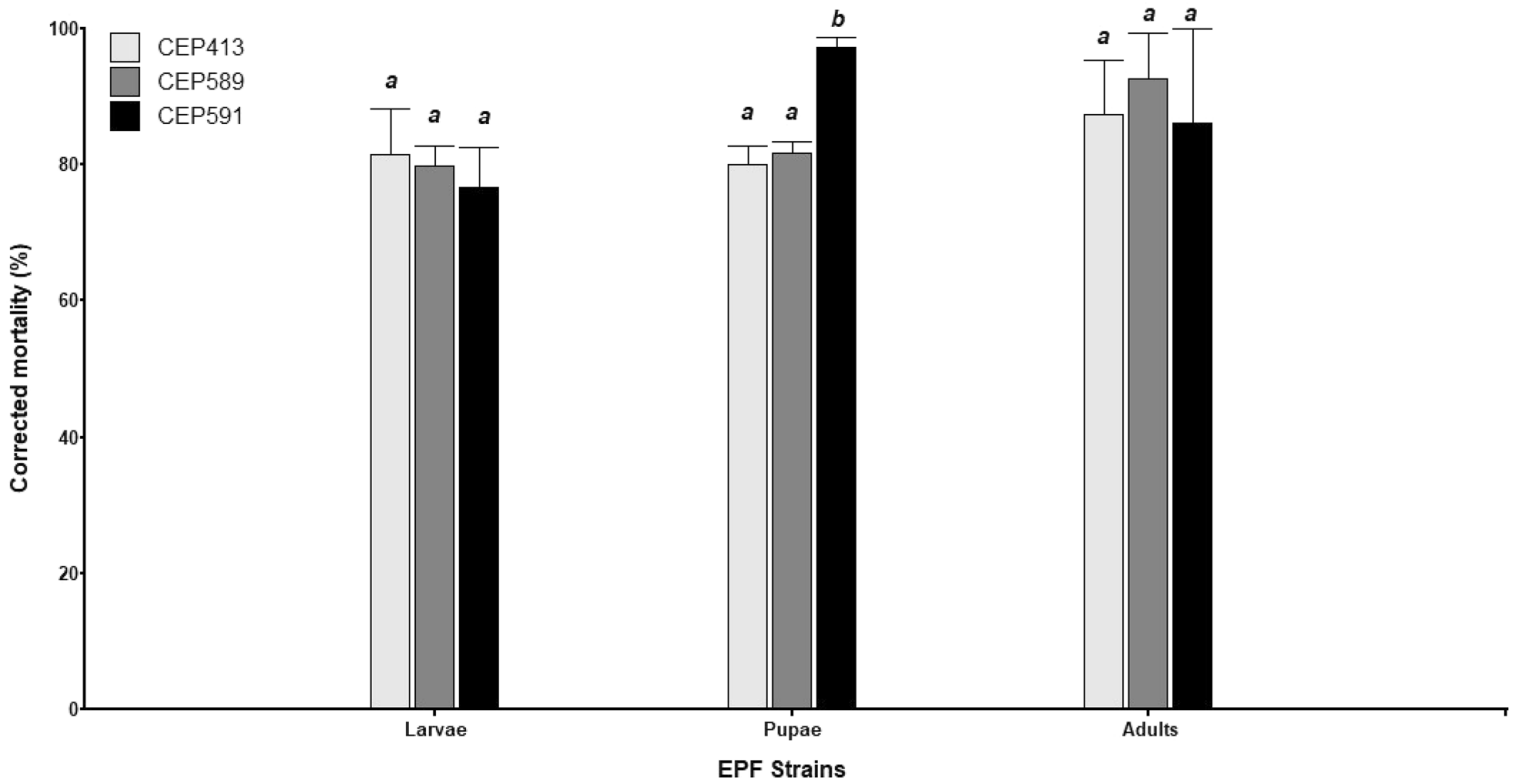
Corrected mortality (%) of *Lobesia botrana* larvae, pupae and adults caused by the three strains of *Metarhizium anisopliae* (CEP413, CEP589, CEP591). Mean percentages ± SD (standard deviation) of infected individuals is indicated for three replicates. Different letters indicate significant differences among treatments within each *L. botrana* stage (Fisher’s LSD; *p* < 0.05)

All *M. anisopliae* strains tested in this study were able to infect *L. botrana* at any developmental stage, including larvae (L_5_), pupae (P_p_), and adults (A_d_) (Fig. 1a–c, respectively). No significant differences were observed among EPF strain efficacy for L_5_ (*F* = 0.22, *p* = 0.8097) and A_d_ (*F* = 0.12, *p* = 0.8905). Nevertheless, for P_p_ stage significant differences in EPF strain efficacy were observed (*F* = 33.25, *p* = 0.0006). CEP591 was observed to be more effective (99.9%) than CEP589 (81.6%) and CEP413 (79.98%) in controlling the pupal stage of *L. botrana* (Fig. 2). Overall, the mortality of the control group was lower than 15% in all cases.

### Biological control of EPF on *L. botrana* in the field

At field conditions, larvae of *L. botrana* were susceptible to EPF infection across all seasons. Nevertheless, *L. botrana* mortality was significantly affected by the season (*F* = 6.92, *p* = 0.0074). Overall, larvae of *L. botrana* were more susceptible to CEP591 at early spring (91%) and early summer (81.5%) than in late summer (Table 1). In late summer, CM drastically decreased to 64.9%. Mortality of control larvae was relatively constant across seasons (*F* = 1.11, *p* = 0.3551), which rounded 5%.

**Table 1:** Evaluation of EPF at field conditions

EPF strain	CM (%)—S_1_	CM (%)—S_2_	CM (%)—S_3_
CEP591	91 ± 9.8^a^	81.58 ± 17.53^a^	64.97 ± 6.88^b^
Temperature	22.5 ± 1.2 °C	25.4 ± 2.5 °C	21.7 ± 2.1 °C
RH	52 ± 12.2%	64 ± 8.4%	61 ± 3.9%

Corrected mortality (CM) ± SD (standard deviation) for L_3_–L_4_ larvae of *Lobesia botrana* caused by *Metarhizium anisopliae* strain CEP591 at field conditions. All trials were performed in vivo (*Vitis vinifera* L. cv. Merlot) on an experimental field located in San Juan (Argentina) (31° 58′ 58″ S; 68° 26′ 28″ W). The trial was initiated at early spring (S_1_) and repeated at early summer (S_2_) and late summer (S_3_), comprising a complete vine productive season. Letter’s a, b indicates significant differences between seasons (Fisher’s LSD; *p* < 0.05)

### Inhibitory effects of EPF on *E. microtheca*

**Fig. 3:**
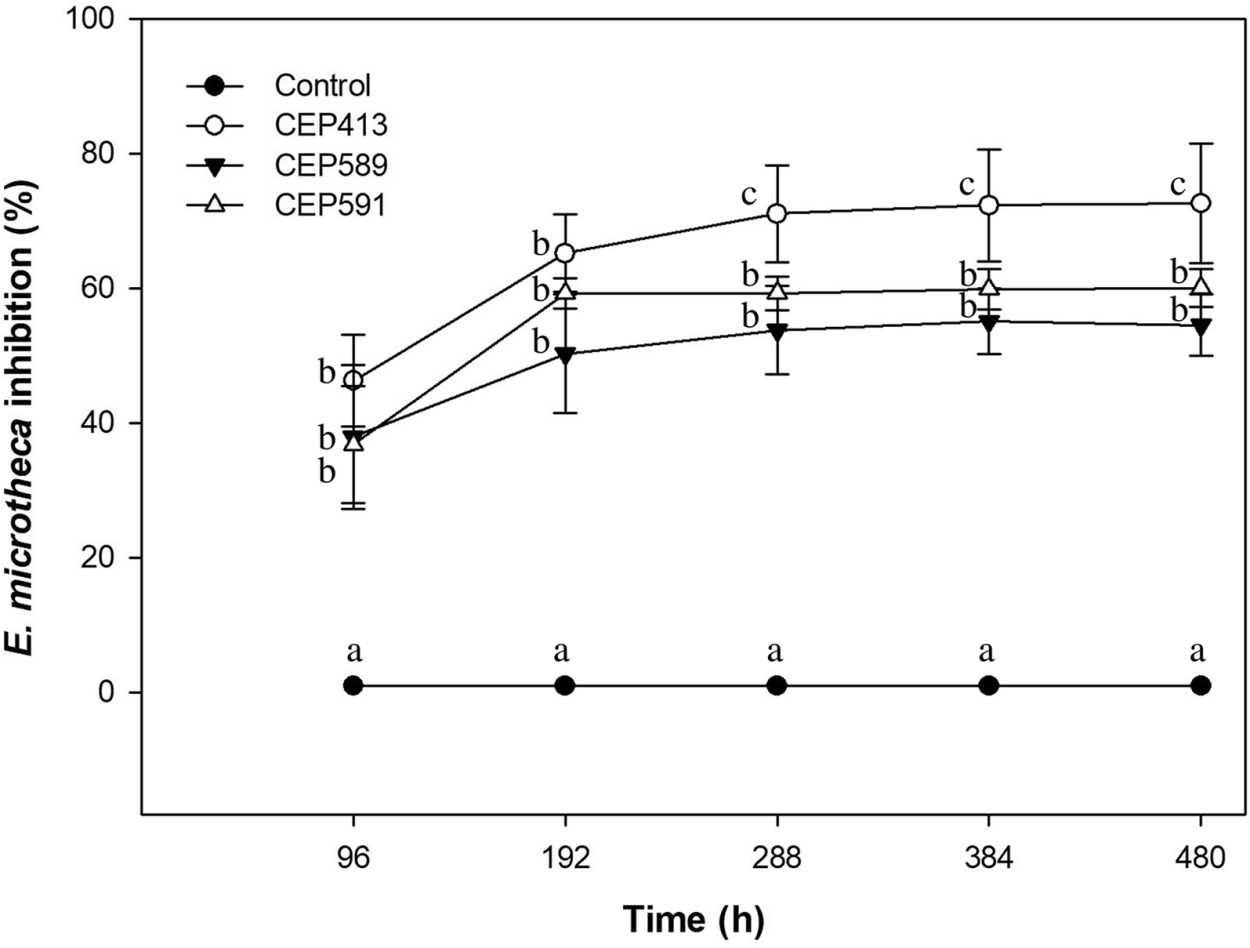
Growth inhibition (%) of the phytopathogenic fungus *Eutypella microtheca* caused by the three strains of *Metarhizium anisopliae* (CEP413, CEP589, CEP591) over 20 days. Control consisted of agar plugs without fungal spores. Mean percentages ± SD (standard deviation) is indicated for three replicates. Different letters indicate significant differences among treatments for each measured time (Fisher’s LSD; *p* < 0.05)

The growth of *E. microtheca* was significantly affected by the EPF strains (*F* = 151.49, *p* < 0.0001). There was a significant decrease in the growth of *E. microtheca* treated with CEP413, CEP589, and CEP591 compared with that of the control group (Fig. 3). The inhibitory percentage of the strain CEP413 (65.2% ± 12.24) was significantly higher than those of CEP591 (55.07% ± 10.11) and CEP589 (50.27% ± 9.12) on *E. microtheca* by the end of the trial (after 480 h) (*F* = 7.8, *p* = 0.0013).

## Discussion

Strains of *M. anisopliae* were highly effective to controlling two detrimental agents of *V. vinifera* L. in vitro and in vivo. EPF strains showed high mortality rates in all developmental stages of *L. botrana* and reduced the growth of the phytopathogenic fungus *E. microtheca*.

Overall, the three *M. anisopliae* strains were highly effective to controlling *L. botrana* at the L_5_, P_p_, and A_d_ stages. The average corrected mortality (CM) of larvae treated with the EPF strains was over 75%, while that of treated pupae and adults was over 85%. However, the CM of pupae treated with CEP591 was significantly higher than that with the other two strains, causing approximately 100% of mortality. This result is particularly important given that the pupal stage is highly resistant to fungal infection, at least in some Lepidoptera [62], Coleoptera [63], and Blattodea [64] species. Consistent with our results, previous studies have also shown the effectiveness of EPF strains in controlling immature stages of *L. botrana*, including larvae and pupae [32]. Similarly, recent findings [65] showed that *Metarhizium* and *Beauveria*, which were isolated from soils under vineyards in Argentina, were effective against the fifth-instar larvae of *L. botrana *in vitro. Additionally, several studies have shown that *Metarhizium* is a successful EPF genus in the biological control of some lepidopteran pests in vitro, such as *Spodoptera litura*, *S. frugiperda*, *Tuta absoluta*, and *Plutella xylostella* [66–69], suggesting that strains of this genus have the potential to be used as biopesticides against several lepidopteran species.

Previous studies on the effectiveness of EPF strains against *L. botrana* have been particularly focused on a single developmental stage of the insect’s life cycle [55, 65, 70, 71]. In this study, we have shown the strong effectiveness of *M. anisopliae* strains against different developmental stages of *L. botrana*, including larvae, pupae, and adults in vitro. These results agree with other studies, which have demonstrated the role of EPF strains as an efficient method controlling all developmental stages of different insect pests such as *Megalurothrips sjostedti* [72], *S. litura* [68], *Dermanyssus gallinae* [73], and *Tetranychus urticae* [74]. Differences in EPF effectiveness between the larval and pupal stage of *L. botrana* may be associated with differences in the ecdysis time between both stages. Larvae, in contrast to pupae, may have shorter time intervals between successive ecdysis, which has been reported to be an important factor in insect resistance to fungal infection [75, 76].

In the field, the strain CEP591 was shown to be effective in killing *L. botrana* larvae across seasons. Nevertheless, efficacy of CEP591 in controlling *L. botrana* in the field was dependent on the season. In late summer, it was observed that CEP591 was less pathogenic to *L. botrana*, causing a mortality of around 64% compared to the other two seasons, in which larval mortality ranged from 81 to 91%. Our results agree with previous results, which have reported a marked decline in the biocontrol efficacy when insect’s pests are challenged by EPF in the field compared to laboratory trials [77–79]. For example, Rodríguez et al. [77] observed that the *M. anisopliae* dose required to kill 50% of *Varroa destructor* (Acari) in vitro was considerably lower (3.8 × 10^5^ c/mL) to that required under natural conditions (5 × 10^10^ c/mL). Similar observations were found by Altimira et al. [55] using the EPF *Beauveria pseudobassiana* to control *L. botrana *in vitro and in vivo. This efficacy variation of EPF in vitro versus in vivo may be associated with abiotic factors, including temperature, relative humidity, and UV radiation, which are known to influence some physiological insect processes such as conidia germination [55, 80, 81].

The results here showed that the three *M. anisopliae* strains were also effective inhibiting the *E. microtheca* growth when co-cultured during 20 days at 28 °C in the dark. The three tested strains significantly inhibited the growth of *E. microtheca* by over 50%. The strain CEP413 was, however, more effective than CEP589 and CEP591 in inhibiting the proliferation and growth of *E. microtheca* over time. Different EPF species, such as *Beauveria bassiana*, *Metarhizium brunneum*, *M. anisopliae*, *Lecanicillium lecanii*, and *Isaria javanica* [33, 34, 82, 83] have been identified as suitable candidates for controlling several phytopathogenic fungi, including *Rhizoctonia solani*, *Pythium myriotylum* [84], *Sphaerotheca fuliginea* [85], and *Botrytis cinerea* [86]. However, to the best of our records this is the first study reporting an antagonistic effect of *M. anisopliae* over *E. microtheca*, showing that *M. anisopliae* strains are also appropriate candidates for use in biological control of one of the most important pathogens of *V. vinifera* L.

## Conclusions

The present study revealed that three strains of *M. anisopliae* were highly effective infecting and killing different developmental stages of *L. botrana *in vitro and in vivo*.* Additionally, the same EPF fungal strains were able to inhibit the growth of the phytopathogen *E. microtheca *in vitro. Our results showed that *M. anisopliae* strains have the potential to be used in a program of integrated pest management aimed to control different detrimental agents of vines. Additional research, however, is required to improve and sustain the efficacy of EPF under natural conditions, particularly under different environmental conditions.

## Data Availability

Figures, and data sheets are available by request to the corresponding author.

## Abbreviations

EPF: Entomopathogenic fungi
IPM: Integrated pest management
MEA: Malt extract agar
PGA: Potato glucose agar
MP: Liquid peptone malt
CM: Corrected mortality
L_5_: Fifth instar larvae
P_p_: Pupae
A_d_: Adults
